# Influenza PA Substitutions and Genetic Diversity of A(H1N1)pdm09, A(H3N2), and B/Victoria Viruses in Japan During the 2023–2024 Season

**DOI:** 10.3390/v18010013

**Published:** 2025-12-21

**Authors:** Nanjun Lee, Julian W. Tang, Irina Chon, Fujio Kakuya, Ryuta Terao, Takashi Kawashima, Isamu Sato, Naoki Kodo, Eitaro Suzuki, Hironori Masaki, Norichika Asoh, Yutaka Shirahige, Hirotsune Hamabata, Tsutomu Tamura, Keita Wagatsuma, Yuyang Sun, Jiaming Li, Tri Bayu Purnama, Yusuke Ichikawa, Hisami Watanabe, Reiko Saito

**Affiliations:** 1 Division of International Health (Public Health), Graduate School of Medical and Dental Sciences, Niigata University, Niigata 951-8510, Japan; irinachon@med.niigata-u.ac.jp (I.C.); waga@med.niigata-u.ac.jp (K.W.); sunyuyang@med.niigata-u.ac.jp (Y.S.); lijiaming@med.niigata-u.ac.jp (J.L.); n23c203c@mail.cc.niigata-u.ac.jp (T.B.P.); n22c202a@mail.cc.niigata-u.ac.jp (Y.I.); jasmine@med.niigata-u.ac.jp (R.S.); 2 Department of Respiratory Sciences, University of Leicester, Leicester LE1 7RH, UK; jwtang49@hotmail.com; 3 Department of Clinical Microbiology, University Hospitals of Leicester NHS Trust, Leicester LE1 5WW, UK; 4 Furano Kyokai Hospital, Furano 076-8765, Japan; f768fk@furano.ne.jp (F.K.); terao-r@asahikawa-med.ac.jp (R.T.); 5 Kawashima Medical Clinic, Shibukawa 377-0008, Japan; takasi@mail.gunma.med.or.jp; 6 Yoiko Pediatric Clinic, Niigata 950-0983, Japan; e-yoiko@guitar.ocn.ne.jp; 7 Kodo Pediatrics, Uji 611-0013, Japan; nkodo43@mbox.kyoto-inet.or.jp; 8 Suzuki Pediatric Clinic, Ube 755-0151, Japan; esuzuki@bronze.ocn.ne.jp; 9 Masaki Respiratory Medicine Clinic, Nagasaki 850-0841, Japan; masaki5908clinic@chorus.ocn.ne.jp; 10 Juzenkai Hospital, Nagasaki 852-0812, Japan; ikyoku.asou@juzenkai-hospital.or.jp; 11 Shirahige Clinic, Nagasaki 850-0003, Japan; yutaka@shirahige.org; 12 Awase-Daichi Clinic, Okinawa City 904-2172, Japan; awase555@cello.ocn.ne.jp; 13 Infectious Diseases Research Center of Niigata University in Myanmar (IDRC), Graduate School of Medical and Dental Sciences, Niigata University, Niigata 951-8510, Japan; ttamura@med.niigata-u.ac.jp (T.T.); hwatanabe@med.niigata-u.ac.jp (H.W.); 14 Institute for Research Administration, Niigata University, Niigata 951-8510, Japan

**Keywords:** influenza A(H1N1)pdm09, influenza A(H3N2), influenza B/Victoria, hemagglutinin gene, phylogenetic analysis, vaccine strain mismatch, PA/I38T, antiviral resistance

## Abstract

We characterized influenza A(H1N1)pdm09, A(H3N2), and B/Victoria viruses circulating in Japan during 2023–2024, focusing on lineage placement relative to WHO-recommended vaccine strains and on baloxavir resistance (PA/I38T substitutions). We enrolled 210 outpatients with influenza-like illness across eight clinics in six prefectures (October 2023–September 2024). Of these, 209 had an analyzable pre-treatment respiratory specimen for RT-PCR; hemagglutinin (HA) and neuraminidase (NA) genes were sequenced by next-generation sequencing (NGS). PA/I38T substitutions that confer baloxavir resistance were assessed by cycling-probe RT-PCR, Sanger sequencing, and NGS. HA phylogenies were constructed with global datasets and WHO vaccine reference strains. Of 209 pre-treatment specimens, 181 were influenza-positive (A(H1N1)pdm09 44.2%, A(H3N2) 37.6%, B/Victoria 18.2%); 51 follow-up specimens were collected ≈4–5 days after baloxavir or neuraminidase inhibitor therapy. HA phylogeny placed A(H1N1)pdm09 in clades 5a.2a/5a.2a.1 with predominance of subclade D.2. A(H3N2) clustered exclusively in clade 2a.3a.1 (J lineage, mostly J.1), indicating a mismatch with the season’s A/Darwin/9/2021 vaccine component and supporting the subsequent J-lineage update. All B/Victoria genomes fell within V1A.3a.2 on a C.5 backbone (C.5.1 and C.5.7). No PA/I38T variant was detected in any pre-treatment specimen. Post-baloxavir, PA/I38T emerged in one A(H3N2) case (confirmed by all three methods) and in one B/Victoria case detected by NGS only (minority variant in a low-load sample). NA genes showed no substitutions associated with reduced susceptibility to laninamivir (e.g., E119A, G147E). During 2023–2024, A(H1N1)pdm09 and B/Victoria remained genetically aligned with their vaccine components, whereas A(H3N2) shifted to the J lineage, consistent with the 2024–2025 vaccine update. Although pre-treatment PA/I38T was absent, low-frequency on-therapy selection was observed, including a rare PA/I38T in influenza B/Victoria detected by NGS, suggesting the value of deep sequencing when viral loads are low. These integrated genomic–clinical data support vaccine strain realignment for H3N2 and continued monitoring of baloxavir resistance in outpatient care.

## 1. Introduction

Influenza viruses, particularly types A and B, remain a significant global public health burden, with seasonal outbreaks causing substantial morbidity and mortality. According to the World Health Organization (WHO), annual influenza epidemics affect up to 10% of adults and 20% of children, resulting in 290,000–650,000 respiratory deaths worldwide [1]. The high mutation rate of these viruses leads to antigenic drift within the hemagglutinin (HA) and neuraminidase (NA) genes, resulting in progressive immune escape and necessitating continual adaptation of vaccine formulations [2,3]. HA and NA, being the primary targets of neutralizing antibodies, are central to both host immune protection and vaccine effectiveness (HA only). Amino acid substitutions within major antigenic regions, particularly the globular head of HA, can substantially reduce vaccine-induced immunity [4], making timely vaccine updates imperative [5,6,7].

The coronavirus disease 2019 (COVID-19) pandemic disrupted typical influenza seasonality, largely due to non-pharmaceutical interventions such as masking, school closures, and travel restrictions [8,9]. While these measures significantly reduced the circulation of influenza viruses globally, sporadic outbreaks still occurred in some regions, including Japan. Following the relaxation of these interventions, countries such as Japan reported atypical influenza activity marked by altered timing, subtype dominance, and partial antigenic mismatch with vaccine strains [9,10]. Notably, a resurgence of seasonal influenza was observed during the 2022–2023 season in Hokkaido, with increased pediatric hospitalizations [11]. These changes challenge both vaccine strain selection and public health preparedness and highlight the renewed importance of robust influenza surveillance systems [12,13].

Beyond vaccination, antiviral agents serve as an adjunct to reduce disease burden. Baloxavir marboxil, an endonuclease inhibitor approved in Japan since 2018, has been widely used among pediatric patients. However, amino acid substitutions at position I38 of the polymerase acidic (PA) protein, particularly I38T and I38M, have been associated with reduced baloxavir susceptibility [14,15]. Emerging evidence suggests that these substitutions occur more frequently in pediatric populations. In a clinical study involving Japanese children aged 1–11 years, PA/I38T or I38M variants emerged in 23.4% of baloxavir-treated patients, and their presence was associated with delayed viral clearance and longer illness duration [16]. Similar age-related patterns were observed in younger children under six years, where transient viral rebound and symptom recurrence were more pronounced in those carrying PA/I38X variants [17].

Our previous surveillance study conducted during the 2022–2023 influenza season identified PA/I38X substitutions in 14.8% of A(H3N2) virus samples following baloxavir administration, and in 0.8% of samples collected prior to treatment, suggesting the potential circulation of reduced-susceptibility variants within the community [18]. Notably, several of these variants were detectable only via next-generation sequencing (NGS), underscoring the importance of high-resolution genomic tools in antiviral resistance surveillance.

This study aims to investigate the genetic features of the hemagglutinin (HA) genes of influenza A(H1N1)pdm09, A(H3N2), and B/Victoria viruses circulating in Japan during the 2023–2024 season, with a focus on their antigenic congruence with World Health Organization (WHO)-recommended vaccine strains. These findings highlight the need for enhanced monitoring of antiviral resistance, particularly within pediatric populations, where baloxavir is frequently prescribed. Supporting evidence from both experimental and field studies has demonstrated the superior sensitivity of NGS for detecting PA/I38T variants and has further elucidated their implications for viral fitness and therapeutic responsiveness [19,20,21].

## 2. Materials and Methods

### 2.1. Sample Collection

#### 2.1.1. Patient Enrollment and Treatment

This observational study was conducted during the 2023–2024 influenza season in Japan and enrolled otherwise healthy outpatients presenting with influenza-like illness (ILI), including symptoms such as fever, sore throat, and general malaise.

Patient recruitment took place between October 2023 and September 2024 across eight outpatient clinics situated in six prefectures (Niigata, Gunma, Kyoto, Yamaguchi, Nagasaki, and Okinawa). Eligible individuals were required to attend a participating clinic within 48 h of symptom onset. Influenza infection was confirmed via a rapid antigen detection test (QuickNavi-Flu + COVID™; Denka Co., Ltd., Tokyo, Japan).

Following diagnosis, antiviral treatment was prescribed at the discretion of each attending physician. One of four antivirals commonly prescribed in Japan, baloxavir, laninamivir, oseltamivir, or zanamivir, was administered. Unless otherwise specified, the date of the outpatient visit was designated as the initiation point of antiviral treatment. Dosing regimens adhered to standard clinical practice guidelines in Japan [22]. For instance, baloxavir was prescribed as a single oral dose of 40 mg for patients aged 12 to 18 years, while weight-based dosing (ranging from 10 to 40 mg) was applied for those under 12 years of age.

#### 2.1.2. Collection of Specimens and Clinical Data

Nasopharyngeal swabs or aspirates were collected at two time points: during the initial visit (prior to antiviral treatment) and again approximately 4–5 days later, a timeframe during which residual viral RNA may still be detectable. At each visit, two swabs were obtained: one was used immediately for rapid diagnostic testing (RDT), and the other was preserved for later molecular analysis. The latter specimens were stored in viral transport media at −20 °C and subsequently shipped to Niigata University for further analysis. In cases where the follow-up sample could not be obtained, only the baseline sample was analyzed.

Demographic and clinical information—including age, sex, vaccination history, time between symptom onset and clinic visit, body temperature, and treatment prescribed was recorded when patients or their guardians consented to maintain a home log. Caregivers were instructed to log axillary temperatures three times daily and report symptom severity across seven categories (e.g., cough, sore throat, muscle pain) using a standardized rating scale for up to eight days. In cases of incomplete reporting, physician observations were used instead. Fever duration was defined from the time of treatment initiation to the last recorded temperature ≥ 37.5 °C. Total symptom duration was determined as the time until complete symptom resolution.

### 2.2. Cycling Probe Real-Time PCR Assay for PA/I38T Screening

Total viral RNA was extracted from 140 μL of clinical specimen using the QIAamp Viral RNA Mini Kit (QIAGEN, Hilden, Germany), and reverse transcribed into complementary DNA (cDNA). The cDNA was analyzed using a department-developed cycling probe real-time PCR assay, designed to identify PA/I38T substitutions in influenza A(H1N1)pdm09 and A(H3N2) viruses. For B/Victoria lineage viruses, a novel primer/probe set was designed to enable screening of PA/I38T. The primer and probe sequences, including target genomic positions, are provided in Table S1.

### 2.3. Sanger Sequencing for PA/I38T Substitution Confirmation

Sanger sequencing was employed to supplement the sequence result of Next-Generation Sequencing. PA gene segments of A(H1N1)pdm09, A(H3N2), and B/Victoria viruses were amplified via nested PCR using primer sets outlined in Table S2. PCR products were purified and sequenced using a BigDye Terminator v3.1 kit (Applied Biosystems, Waltham, MA, USA) and analyzed with a SeqStudio™ Genetic Analyzer (ThermoFisher Scientific, Waltham, MA, USA). Resulting sequences were aligned to reference strains A/Brisbane/02/2018, A/Kansas/14/2017, and B/Colorado/06/2017 to identify amino acid substitutions at key positions (e.g., 23, 37, 38, and 119) in PA gene associated with baloxavir resistance, as recommended by WHO guidelines [23]. Sequence alignments were performed using BioEdit v7.7.1 [24].

### 2.4. Genetic Analysis by Next-Generation Sequencing

Samples with a cycle threshold (C*_t_*) ≤ 32 and selected across various months were prioritized for NGS. Reverse transcription and amplification were performed using a one-step Superscript III RT-PCR kit (Invitrogen, Waltham, MA, USA) [25]. Amplicons were purified, quantified, and prepared into sequencing libraries following established protocols [26,27]. Sequencing was carried out on the Illumina iSeq 100 platform (Illumina, San Diego, CA, USA).

Paired-end reads (~250 bp) were processed and assembled using CLC Genomics Workbench v24.0.1 (CLC bio, Cambridge, MA, USA). Assembled viral genomes were aligned to representative reference strains: A/H1N1/Illinois/08/2018 (GenBank: MH727752- MH727759), A/H3N2/Louisiana/50/2017 (CY244750–CY244757), and B/Victoria/Washington/02/2019 (MN155748–MN155755). Genetic analyses were conducted across all eight gene segments based on established protocols [27]. The assembled viral genomes were further analyzed to detect amino acid substitutions related to antiviral resistance. Substitution calling was performed using CLC Genomics Workbench tools, allowing quantification of substitution allele frequencies. The PA gene segment was screened for substitutions potentially associated with reduced baloxavir susceptibility—such as E23G/K, K34R, A37T, I38F/L/M/S/T/V, and E199G—based on WHO guidelines [23].

In parallel, the NA gene was examined for substitutions conferring resistance to neuraminidase inhibitors (NAIs), including E119D/I/V, I222L, R224K, H275Y, R292K, N294S, and S331R [28]. A threshold of ≥5% allele frequency and minimum read depth of 100 was applied for variant calling, in accordance with established practices [29].

### 2.5. Subclade Classification by Phylogenetic Tree

Subclade classification was performed through phylogenetic analysis of the HA gene sequences. Vaccine and reference strains were selected and downloaded based on the WHO recommendations [30] and the annual and Interim report from the Crick Worldwide Influenza Centre (WIC) (London, UK) [31]. The representative vaccine strains for Japan during the 2023–2024 and 2024–2025 seasons included A/Victoria/4897/2022 Egg IVR-238 (EPI ISL 18557516) for A(H1N1)pdm09, A/Darwin/9/2021 SAN-010 (EPI ISL 17352397) and A/California/122/2022 SAN-022 (EPI ISL 18608328) for A(H3N2), and B/Australia/1359417/2021 Egg BVR-26 (EPI ISL 5196190) for B/Victoria lineage.

Additional reference strains were selected from the Global Initiative on Sharing All Influenza Data (GISAID) database based on high sequence similarity to the study samples, identified using the BLAST (BLAST+ 2.17.0) search tool implemented in GISAID. All strains were retrieved from the GISAID platform (https://gisaid.org; accessed on 13 November 2025) and were collected from global surveillance between January 2023 and May 2024.

Phylogenetic trees for A(H1N1)pdm09, A(H3N2), and B/Victoria were constructed using the maximum likelihood method implemented in MEGA software v6.0.6 (USA, Texas) [32]. Model selection was based on the lowest Akaike information criterion (AIC) value. Bootstrap analysis with 1000 replicates was used to assess tree reliability.

### 2.6. GISAID Registration

The consensus HA and NA sequences generated in this study have been deposited in the GISAID EpiFlu database. The registration numbers are as follows:

A(H1N1)pdm09: EPI_ISL_20180921, EPI_ISL_20180959, EPI_ISL_20180960, EPI_ISL_20180966–EPI_ISL_20180985, EPI_ISL_20182574–EPI_ISL_20182576, EPI_ISL_20182578–EPI_ISL_20182582, EPI_ISL_20182584, EPI_ISL_20182585, EPI_ISL_20182587–EPI_ISL_20182593, EPI_ISL_20182596, EPI_ISL_20182597, EPI_ISL_20195340 and EPI_ISL_20195341.

A(H3N2): EPI_ISL_20184088, EPI_ISL_20184090, EPI_ISL_20184238, EPI_ISL_20184245-EPI_ISL_20184247, EPI_ISL_20184250, and EPI_ISL_20184259- EPI_ISL_20184284; B/Victoria: EPI_ISL_20181505–EPI_ISL_20181528, EPI_ISL_20195342 and EPI_ISL_20195343.

B/Victoria: EPI_ISL_20181505–EPI_ISL_20181528, EPI_ISL_20195342 and EPI_ISL_20195343.

## 3. Results

### 3.1. Sample Collection, Subtype Distribution, and Seasonal Trends During the 2023–2024 Influenza Season in Japan

**Figure 1:**
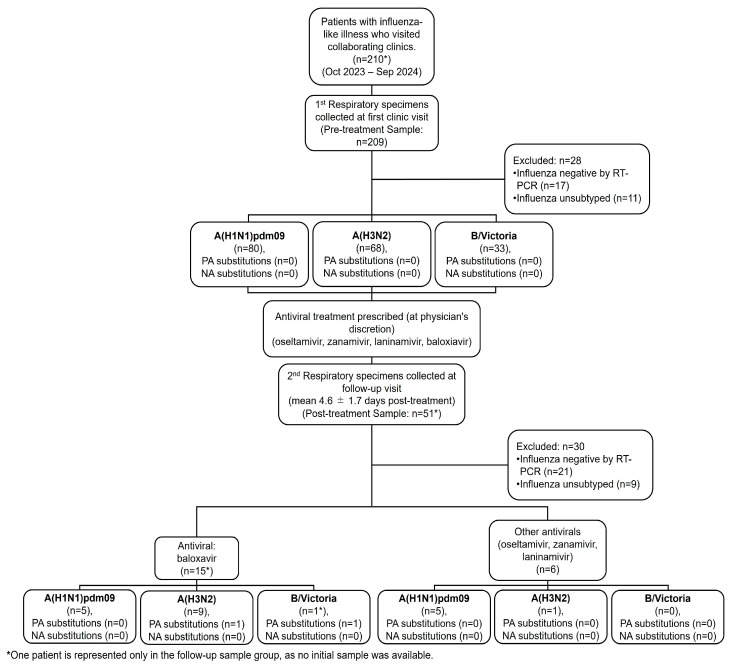
Flow chart for patient selection. Patients with influenza-like illness were enrolled Oct 2023–Sep 2024 (*n* = 210). Pre-treatment samples at first visit (*n* = 209) yielded 181 subtyped after exclusions A(H1N1)pdm09 80; A(H3N2) 68; B/Victoria 33. Follow-up samples 4.6 ± 1.7 days post-therapy (*n* = 51) left 21 for analysis (baloxavir 15; other antivirals 6). Boxes indicate subtype/lineage and counts of PA and NA substitutions. One B/Victoria case lacked a pre-treatment sample.

Respiratory samples were collected from 210 outpatients presenting with influenza-like illness between October 2023 and September 2024 (Figure 1). A total of 209 samples were obtained at the initial clinic visit, prior to the initiation of antiviral therapy. An additional 51 follow-up samples were collected approximately 4–5 days after treatment from patients who returned for follow-up visits. Among these 51 follow-up samples, one was from a patient for whom no pre-treatment sample was available. The choice of antiviral agent, including baloxavir, oseltamivir, zanamivir, laninamivir, or peramivir, was made at the discretion of the attending physician during the initial consultation.

Age and sex data were available for 203 (96.7%) of the 210 enrolled patients. The median age was 9.8 years (interquartile range [IQR], 6.2–13.3 years), and ages ranged from 0 to 93.6 years. Among these 203 patients, children < 15 years accounted for 83.7% (170/203), those aged 15–64 years for 14.8% (30/203), and those aged ≥65 years for 1.5% (3/203). Overall, 117 (57.6%) were male and 86 (42.4%) were female. Vaccination history for the 2023–2024 influenza season was available for 178 patients; among these, 39 (21.9%) had received at least one dose of the seasonal influenza vaccine, whereas 139 (78.1%) had not been vaccinated. Among 186 patients with known dates and times of fever onset and clinic visit, the median interval from symptom onset to clinic presentation was 1.0 day (interquartile range [IQR], 0.6–1.1 days). Baseline body temperature at the initial visit was available for 152 patients, with a median of 38.9 °C (IQR, 38.3–39.4 °C).

Among the 209 pre-treatment samples, 17 were excluded due to being influenza-negative by RT-PCR and 11 were unsubtyped, leaving 181 samples for subtype analysis. Of these, 80 (44.2%) were identified as A(H1N1)pdm09, 68 (37.6%) as A(H3N2), and 33 (18.2%) as B/Victoria. Of the 51 post-treatment samples, 21 were excluded due to negative RT-PCR results and 9 were unsubtyped, leaving 21 samples available for subtype analysis. Among these 21, 15 samples were from patients who had received baloxavir treatment. Of these, 5 samples were identified as A(H1N1)pdm09, 9 samples as A(H3N2), and 1 sample as B/Victoria. This B/Victoria patient is included only in the follow-up sample group, as no initial sample was available. The remaining 6 samples were from patients treated with other antivirals, with 5 samples identified as A(H1N1)pdm09 and 1 sample as A(H3N2).

**Figure 2:**
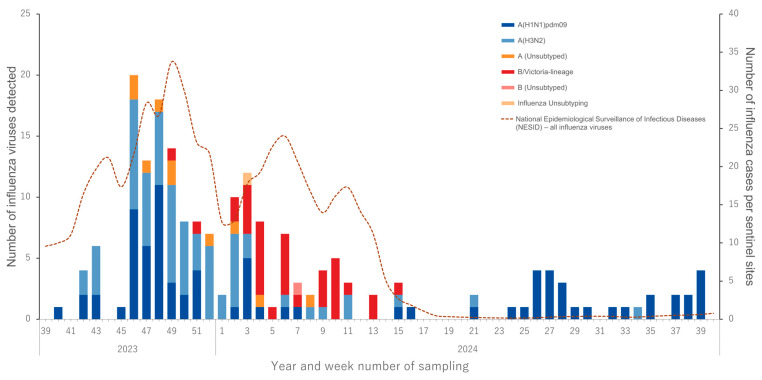
Influenza virus detections during the 2023–2024 season confirmed by RT-PCR. Bars represent the number of influenza-positive respiratory samples in this study, categorized by subtype or lineage (dark blue: A(H1N1)pdm09; light blue: A(H3N2); red: B/Victoria; orange: unsubtyped A; pink: unsubtyped B; beige: unsubtyped influenza). The dashed line represents national influenza surveillance data (NESID) (https://id-info.jihs.go.jp/surveillance/idwr/rapid/sokuhou.html, accessed on 13 November 2025)), showing the total number of influenza cases per sentinel site—including all virus types and subtypes—plotted on the right-hand (RH) axis.

The 2023–2024 influenza season in Japan commenced in late October (around epidemiological week 43) and peaked in late November (week 47), followed by a gradual decline extending into mid-March (week 11 of 2024) (Figure 2). The season was characterized by the co-circulation of influenza A and B viruses. A(H1N1)pdm09 was the predominant subtype during the early peak in November.

From December onwards, B/Victoria viruses showed a steady increase and became the dominant strain between January (week 3) and early March. In contrast, A(H3N2) viruses were detected only sporadically and did not predominate at any point during the season. Following March, influenza activity persisted at low levels, with intermittent detection of A(H1N1)pdm09 continuing throughout the summer and into early autumn, up to at least week 39 of 2024 (Figure 2). This seasonal pattern was consistent with national influenza surveillance data from the National Epidemiological Surveillance of Infectious Diseases (NESID) [33].

For epidemiologic context, Figure 2 overlays the National Epidemiological Surveillance of Infectious Diseases (NESID)—Japan’s nationwide sentinel-based system that compiles weekly influenza case reports from 5000 designated clinics and hospitals (broken line, cases per sentinel site) [34]. The temporal pattern of our detections closely paralleled the NESID curve, indicating that our sampling reflected national trends.

### 3.2. Genetic and Phylogenetic Characteristics of Influenza Viruses in 2023–2024 Season

#### 3.2.1. A(H1N1)pdm09 Viruses

**Figure 3:**
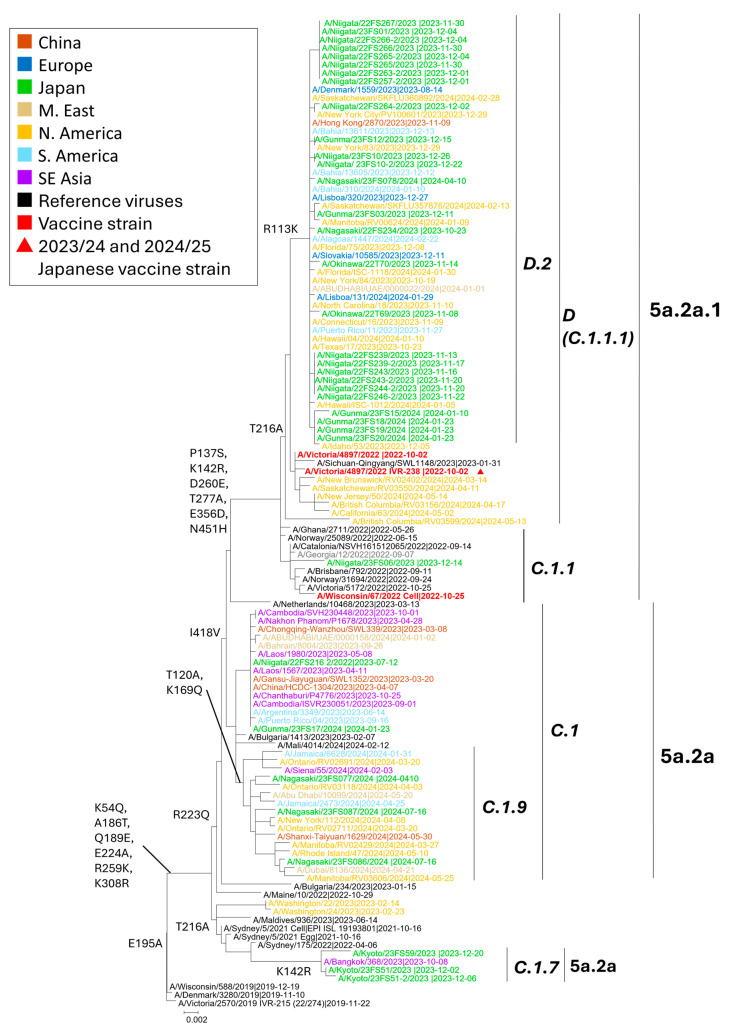
Maximum-likelihood phylogeny of influenza A(H1N1)pdm09 HA sequences collected from October 2023 to September 2024. Tips are color-coded by region of collection (legend). Red triangles denote the Japanese vaccine reference viruses for 2023/24 and 2024/25. Clade and subclade assignments follow WHO nomenclature: 6B.1A.5a.2 and 6B.1A.5a.2a.1 with subclades C.1, C.1.7, C.1.9, C.1.1, and D (including D.2; formerly C.1.1.1). Reference strains used for placement are included. Key branch-defining HA substitutions are annotated on internal nodes. Node support values are from 1000 bootstrap replicates.

Among the 36 A(H1N1)pdm09 samples analyzed by NGS, HA phylogeny placed all sequences within clades 5a.2a and 5a.2a.1. Within 5a.2a, we identified subclades C.1, C.1.7, and C.1.9, each defined by the WHO-designated signature amino-acid substitutions (Figure 3) [35,36]. Within 5a.2a.1, viruses fell into subclades C.1.1 and D.2 (the latter within subclade D, formerly C.1.1.1). The majority of the sequenced A(H1N1)pdm09 viruses clustered in D.2 (27/36; 75.0%). Notably, subclade D is represented by the 2023–2024 vaccine strain A/Victoria/4897/2022. Overall, these viruses were genetically similar to contemporaneous strains circulating in North and South America, Europe, and Southeast Asia.

#### 3.2.2. A(H3N2) Viruses

**Figure 4:**
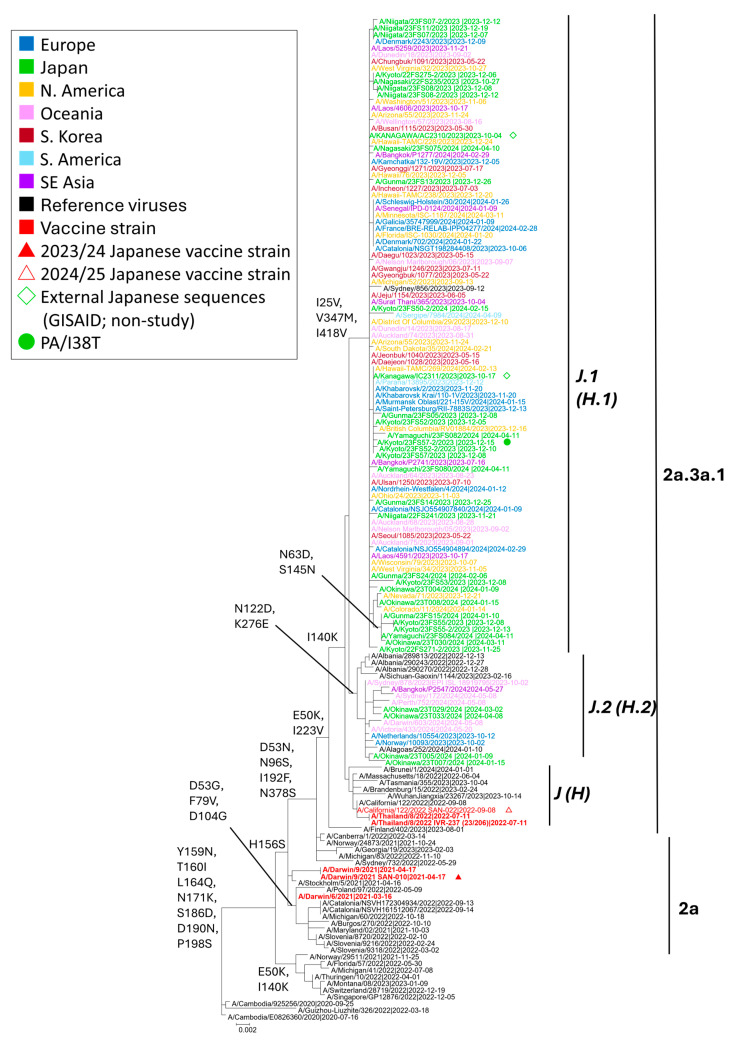
Maximum-likelihood phylogeny of influenza A(H3N2) HA sequences collected from October 2023 to September 2024. Tips are color-coded by region (legend). Red triangles denote the Japanese vaccine reference viruses (filled, 2023/24; open, 2024/25); green diamonds indicate sequences generated by laboratories in Japan, downloaded from GISAID; green circles mark cases in which PA/I38T was detected. Subclade labels follow the late-2024 WHO/Francis Crick nomenclature (J, J.1, J.2), with earlier labels shown in parentheses (H, H.1, H.2). The tree was inferred by the TN93 model with 1000 bootstrap replicates.

Of the 33 A(H3N2) genomes sequenced by NGS, HA phylogeny placed all viruses within clade 2a.3a.1, now termed the J lineage (formerly the H lineage) (Figure 4). Within this lineage, strains segregated into J.1 (formerly H.1) and J.2 (formerly H.2). Subclade J.1 was defined by I25V and V347M (often ± I418V), whereas J.2 carried N122D and K276E [7,35]. The majority (29/33, 87.8%) clustered in J.1, with the remainder in J.2. Relative to the 2023–2024 egg-based vaccine strain A/Darwin/9/2021 (Darwin lineage; 2a/G), all circulating viruses had shifted to the J lineage, consistent with drift during that season. The 2024–2025 update to A/California/122/2022 (2a.3a.1/J), a J-lineage reference, therefore better matches the J.1/J.2 viruses observed in Japan and is expected to improve coverage. Genetically, the Japanese sequences were interspersed with contemporaneous viruses from Europe, Australia/Oceania, North America, and East/Southeast Asia, indicating participation in the global J lineage circulation during the study period.

#### 3.2.3. B/Victoria Viruses

**Figure 5:**
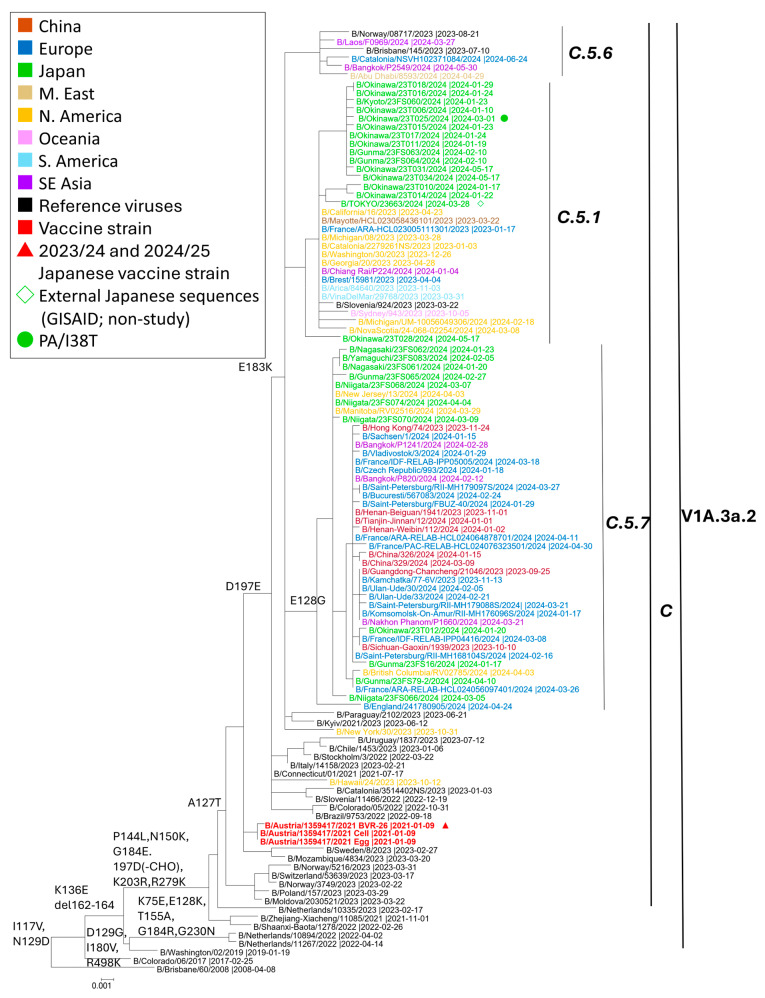
Maximum-likelihood phylogeny of influenza B/Victoria HA sequences collected October 2023–September 2024. Tips are color-coded by region (legend). Open red triangles denote the Japanese vaccine reference strain, B/Austria/1359417/2021 (used in 2023/24 and 2024/25). Green diamonds indicate sequences generated by other laboratories in Japan, downloaded from GISAID; green circles mark a case with PA/I38T detected. Subclade labels follow current WHO/Francis Crick nomenclature: viruses fall within V1A.3a.2 on a C backbone, and only C.5.1 and C.5.7 were detected in this study. The tree was inferred under the GTR + G + I model with 1000 bootstrap replicates.

All 26 B/Victoria genomes sequenced by NGS in Japan belonged to V1A.3a.2 on a C.5 backbone (Figure 5) [35,36]. Within C.5, only two subclades were detected: C.5.1 and C.5.7. C.5.1 comprised 14/26 strains (~54%), and C.5.7 comprised 12/26 (~46%). As expected for C.5 descendants, sequences carried D197E in the HA 190-helix; subclade-defining changes were E183K (C.5.1) and E128G + E183K (C.5.7; 120-loop and site-B vicinity). The vaccine strain for the 2023–2024 season, B/Austria/1359417/2021, is positioned upstream within V1A.3a.2 subclade C (not within C.5). Japanese sequences intermingled with contemporaneous viruses from Europe and North America, indicating participation in the same C.5-derived lineages during the study period.

### 3.3. Frequency of PA Substitution Emergence

Among the 181 pre-treatment samples analyzed by both RT-PCR and NGS, no PA/I38T substitutions were detected in influenza A(H1N1)pdm09 (*n* = 80), A(H3N2) (*n* = 68), or B/Victoria (*n* = 33). This suggests that such substitutions were absent in the patient population prior to antiviral administration.

Following baloxavir treatment, 35 post-treatment samples were tested using both RT-PCR and NGS. One A(H3N2) virus (1/14, 7.1%) tested positive for PA/I38T using RT-PCR, and the result was subsequently confirmed by NGS. In contrast, no PA/I38T substitutions were identified in post-treatment A(H1N1)pdm09 (*n* = 17) samples using either method. One B/Victoria virus (25.0%) was detected to carry a PA substitution by NGS, while RT-PCR was negative due to low viral load (Table 1).

To further clarify the frequency of baloxavir-induced resistance, we assessed a subset of 15 samples that remained RT-PCR or NGS-positive after treatment. Within this subset, the detection rate of PA/I38T increased to 11.1% (1/9) in A(H3N2) and 100% (1/1) in B/Victoria viruses, again confirmed solely by NGS.

### 3.4. Emergence of PA Substitution After Baloxavir Treatment

During the study period, two cases possessing PA/I38T substitution were detected following baloxavir treatment.

One patient was infected with A(H3N2) and was a previously healthy, vaccinated 10-year-old. The patient developed a high fever of 39.0–39.6 °C on the previous day before the clinic visits, with the onset of sore throat, headache, and chill. In the morning before the clinic visit, the temperature decreased to 37.2 °C, with persistence of cough, rhinorrhea, severe headache, and chill (Table 2). After RDT showed positive for influenza A, the clinician prescribed baloxavir as an antiviral treatment. The pre-treatment specimen collected at the first visit showed wild-type PA I38 only by cycling-probe RT-PCR, and NGS confirmed I:100%/T:0%. A follow-up specimen collected 4 days later demonstrated emergence of PA I38T: RT-PCR detected I38T and Sanger sequencing confirmed this result; NGS estimated T:87.9% and I:12.1% (Table 2). The patient’s fever resolved within 23.3 h; however, other symptoms persisted for a total duration of 4.0 days after baloxavir treatment initiation.

The second PA/I38T case involved a 13-year-old with a biphasic clinical course of influenza B/Victoria. Two weeks before the first episode, the patient’s 6- and 10-year-old siblings had influenza B and recovered after baloxavir (Figure S1). The patient developed a fever on 6 February 2024 (peak 39.3 °C) and was seen the next day; the RDT was negative, and no specimen was taken, but household exposure could not be excluded, so baloxavir 40 mg was prescribed empirically. Symptoms improved that day, and oral fluids were tolerated (Table 2; Figure S1). About one week later (15–16 February), a second, more severe febrile episode occurred with headache, nausea, and loss of appetite; the RDT was positive for influenza B, and a second 40 mg dose of baloxavir was given with clinical improvement (Figure S1). A respiratory specimen obtained during this second episode was negative by cycling-probe RT-PCR for influenza B (consistent with low viral load), but NGS detected a mixed population with PA/I38T at 61.1%, whereas Sanger sequencing identified only wild-type PA gene alignment of influenza B/Victoria (Table 2), consistent with minority-variant detection limits at low template. Notably, PA/I38T in influenza B/Victoria clinical samples is rare, and based on available surveillance summaries, has not been previously reported [37,38].

This case underscores the added value of deep sequencing for resistance detection at low viral loads. Because no specimen was collected at the first episode and no additional respiratory pathogen testing was performed, the sequence of events remains uncertain: the second episode may have marked the onset of influenza B/Victoria (so-called superinfection, with the first episode due to another pathogen), or both episodes may reflect a single, through-course influenza B/Victoria infection, with late/convalescent sampling explaining the low viral load at the second visit.

## 4. Discussion

In this study, we investigated the evolutionary dynamics of seasonal influenza viruses circulating in Japan during the 2023–2024 season, with a dual focus on (I) lineage turnover and vaccine fit and (II) antiviral resistance, particularly baloxavir-associated PA/I38T variants.

### 4.1. Molecular Epidemiology of Circulating Influenza A(H1N1)pdm09, A(H3N2) and B/Victoria Viruses

Since the antigenic properties of influenza viruses are largely determined by the hemagglutinin (HA) glycoprotein, HA gene analysis is a primary lens for inferring antigenic relatedness, and thus vaccine match or mismatch.

NGS and phylogenetic analyses placed all A(H1N1)pdm09 viruses collected in Japan within clades 5a.2a and 5a.2a.1, with subclade D.2 constituting the majority. This pattern aligns with global surveillance: since early 2024, C.1.9 has predominated worldwide, whereas D-lineages have remained relatively common in parts of the Americas [35,36]. Within the D-lineage, D.2 exhibits a geographically restricted distribution, primarily reported in Japan and China, with sporadic detection in the United States. In contrast, C.1.1 is a minor lineage and D.1/D.3/D.4 circulate at only low levels.

Serologically, post-vaccination antisera to A/Victoria/4897/2022 (egg-based) and A/Wisconsin/67/2022 (cell-based) demonstrated good reactivity against recent H1N1pdm viruses, with no consistent four-fold HI titer reductions for the egg-based reagent. These findings, together with D.2 predominance, support good antigenic matching of the H1 component used in Japan during the study period, though continued monitoring of expanding 5a.2a.1 (D-lineage) viruses remain warranted.

All Japanese A(H3N2) sequences belonged to subclade 2a.3a.1/J, dominated by J.1 (I25V, V347M ± I418V) and a smaller proportion of J.2 (N122D, K276E). This genetic placement is downstream of the Darwin lineage (2a/G), explaining the phylogenetic separation from the 2023–2024 vaccine reference. In parallel, the February 2024 WHO report described global dominance of J-lineage 2a.3a.1 viruses since late 2023, with J.2 prevalent across Europe, North America, Oceania, and West/South Asia, and J.1 more common in Africa, East Asia, and South America [6].

Antigenic analyses by WHO and collaborating centers indicated reduced reactivity of Darwin-like antisera against subsets of 2a.3a.1 viruses, whereas antisera raised against 2a.3a.1 references (e.g., A/California/122/2022-like) displayed improved recognition of contemporary strains [6,7,35,36]. These findings support the recommendation of J-lineage strains for the 2024–2025 vaccine composition.

B/Victoria viruses detected in Japan were classified within clade V1A.3a.2/C.5, specifically subclades C.5.1 and C.5.7 (≈54% and ≈46%, respectively). This distribution mirrors contemporaneous global observations. Reports from the Francis Crick Institute and WHO similarly identified C.5-derived lineages as predominant worldwide, with C.5.1 prevailing in the Americas and parts of Europe, and C.5.7 prominent across East Asia, China, Australia, and Eurasia [6,7,35,36]. Antigenically, V1A.3a.2 viruses were well recognized by B/Austria/1359417/2021-like antisera, despite accumulating substitutions in antigenic regions (E128G, E183K, D197E).

### 4.2. Antiviral Resistance and Clinical Interpretation

In general, interpretation of antiviral resistance requires distinguishing between pre-treatment and post-treatment findings: pre-treatment variants represent circulating resistance with public health implications, whereas post-treatment variants reflect on-treatment selection and impact primarily individual clinical outcomes.

No PA/I38T substitutions were identified in any pre-treatment sample (A(H1N1)pdm09 *n* = 17; A(H3N2) *n* = 44; B/Victoria *n* = 23) in this study. These results align with nationwide Japanese anti-influenza drug resistance surveillance, which reported low baseline prevalence during 2023–2024 (≈0.4% A(H1N1)pdm09, ≈0.5% A(H3N2), 0% B/Victoria) [39], and with WHO summaries indicating ≤0.12% global prevalence without evidence of sustained transmission [37].

Among post-treatment samples, PA/I38T was detected in 7.1% of A(H3N2) cases (1/14), 0% of A(H1N1)pdm09 (0/17), and 25.0% of B/Victoria cases (1/4). The A(H3N2) mutation was confirmed by cycling-probe RT-PCR, Sanger sequencing, and NGS, whereas the B/Victoria mutation was detected only by NGS as a minority variant, highlighting the superior sensitivity of deep sequencing in low-titer samples.

These post-treatment PA/I38X frequencies are consistent with phase-3 trial results (~3–11% in adults and >23% in children, typically higher in A(H3N2) than A(H1N1)pdm09 and rare in influenza B) [14,16,40], and they align with prior Japanese observational studies—including our own—reporting ~9–15% emergence in A(H3N2) [18,41,42,43]. Clinically, the A(H3N2) PA/I38T case in our cohort did not exhibit prolonged fever or delayed recovery compared with wild-type infections, consistent with previous pediatric observations [43,44].

In this study, we identified a rare PA/I38T substitution in an influenza B/Victoria virus from a patient. Across the WHO Antiviral Working Group updates by Takashita et al., Govorkova et al. and Hussain et al., PA resistance markers in influenza B have been very uncommon: occasional PA-I38V or PA-M34I were noted, but clinical detections of PA/I38T in influenza B have not been documented, and no onward transmission has been reported [37,38,45]. One analysis flagged a B/Yamagata I38T sequence without phenotypic confirmation, underscoring how rare this substitution is in B viruses [37]. Experimental work helps explain its scarcity: recombinant B/Victoria or B/Yamagata viruses carrying PA/I38T show reduced baloxavir susceptibility but clear fitness costs—diminished polymerase activity, impaired replication, and poor competitiveness in animals [46]. In ferret models, baloxavir treatment curtailed influenza B transmission, and I38T-bearing B viruses transmitted less efficiently than wild type, suggesting limited epidemic potential even when the mutation arises under drug pressure [47]. Collectively, global surveillance and experimental data indicate that PA/I38T in influenza B remains exceedingly rare and biologically constrained—consistent with our finding of a low-frequency, post-treatment I38T minority variant detectable only by deep sequencing—supporting continued baloxavir use alongside NGS-based resistance surveillance [37,38,45,47].

Laninamivir was prescribed to 8.1% (17/209) of outpatients in this cohort, a lower proportion than nationwide estimates due to preferential sampling of baloxavir-treated patients. National prescription data for 2023–2024 showed the highest use for oseltamivir, followed by laninamivir, baloxavir, zanamivir, and peramivir (Figure S2) [48]. No NA substitutions associated with reduced laninamivir susceptibility (e.g., E119A, G147E) were detected [49].

### 4.3. Limitation

This study has several limitations. First, the sample size, limited number of participating clinics, and outpatient-based sampling may not fully capture nationwide circulation patterns or severe cases requiring hospitalization. Second, follow-up timing (~4–5 days post-treatment) and Ct-based selection for NGS may bias analyses toward higher-viral-load specimens and under-detect low-frequency variants. Third, one B/Victoria case lacked a pre-treatment specimen, preventing confirmation of de novo emergence. Fourth, phenotypic assays (e.g., endonuclease inhibition, NA inhibition, HA antigenic characterization) were not performed, and antigenic interpretations rely on external data. Finally, detection of minority variants is influenced by sequencing depth and thresholds; discrepancies between Sanger and NGS in low-template samples underscore the risk of false negatives from targeted assays and stochastic variation in low-frequency variant calling.

## 5. Conclusions

In Japan’s 2023–2024 season, A(H1N1)pdm09 and B/Victoria remained genetically close to the corresponding vaccine components, whereas A(H3N2) shifted to the 2a.3a.1 (J) lineage, consistent with the subsequent vaccine update. Pre-treatment PA/I38T was not detected, but post-baloxavir I38T emerged in two cases—one A(H3N2) (confirmed by multiple methods) and one B/Victoria (NGS-only)—suggesting low-frequency, on-therapy selection and underscoring the added value of deep sequencing when viral loads are low. Together, these findings support continued genomic surveillance integrated with clinical and resistance testing to guide vaccine strain selection and antiviral stewardship in routine outpatient care.

## Figures and Tables

**Table 1:** Frequency of PA/I38T substitution during 2023–2024 season by three methods.

Drug Treatment and Detection Method	Pre-Treatment			Post-Treatment with Baloxavir					
				All Patients Receiving Baloxavir			Patients Eligible for RT-PCR or NGS After Receiving Baloxavir		
Subtype and Lineage	Analyzed by RT-PCR and NGS (*n* = 181)	PA Substitution by RT-PCR (%)	NGS (%)	Analyzed by RT-PCR and NGS (*n* = 35)	PA Substitution by RT-PCR (%)	NGS (%)	Analyzed by RT-PCR and NGS (*n* = 15)	PA Substitution by RT-PCR (%)	NGS (%)
A(H1N1)pdm09	80	0 (0.0%)	0 (0.0%)	17	0 (0.0%)	0 (0.0%)	5	0 (0.0%)	0 (0.0%)
A(H3N2)	68	0 (0.0%)	0 (0.0%)	14	1 (7.1%)	1 (7.1%)	9	1 (11.1%)	1 (11.1%)
B/Victoria	33	0 (0.0%)	0 (0.0%)	4	0 (0.0%)	1 (25.0%)	1 *	0 (0.0%) *	1 (100.0%) *

* One B/Victoria case lacked a pre-treatment sample. A follow-up sample collected after baloxavir treatment was RT-PCR–negative but yielded sequence data by NGS, which identified PA/I38T at a variant frequency of 61.1%. This case is excluded from the pre-treatment group and included only in the post-treatment (baloxavir) group.

**Table 2:** Clinical and virological characteristics of the patient who developed PA/I38T substitution after receiving baloxavir.

Clinical and Laboratory Information	Patient 1	Patient 2
Subtype/lineage	A (H3N2)	B/Victoria
Amino acid substitution in PA	I38T	I38T
Clinical information		
Age	10.0 years	13.3 years
Influenza vaccination	Yes	No
Date of onset	10 December 2023	6 February 2024
Time from onset to first clinic visit	26 h	24 h
Fever at the first visit	Not recorded	39.3 °C
Fever duration	23.3 h	Not recorded
Symptom duration	4.0 days	Not recorded
1st visit sample	A/Kyoto/23FS57/2023	Not collected
Cycling Probe RT-PCR	I38 (FAM)	n/a
C*_t_* value	33.8	n/a
Sanger sequencing	I38	n/a
Proportion of variant by NGS	I: 100%, T: 0.0%	n/a
2nd visit sample	A/Kyoto/23FS57-2/2023	B/Okinawa/23T025/2023
Interval from 1st to 2nd	4 days	n/a
Cycling Probe RT-PCR	I38T (ROX)	Negative
C*_t_* value	36.7	n/a
Sanger sequencing	I38T	I38
Proportion of variant by NGS	I: 12.1%, T: 87.9%	I: 38.9%, T: 61.1%

Abbreviations: PA, polymerase acidic protein; NGS, next-generation sequencing; n/a, not applicable.

## Data Availability

The whole-genome sequences generated in this study have been deposited in the Global Initiative on Sharing All Influenza Data (GISAID) EpiFlu database under the following accession numbers: A(H1N1)pdm09: EPI_ISL_20180921, EPI_ISL_20180959, EPI_ISL_20180960, EPI_ISL_20180966–EPI_ISL_20180985, EPI_ISL_20182574–EPI_ISL_20182576, EPI_ISL_20182578–EPI_ISL_20182582, EPI_ISL_20182584, EPI_ISL_20182585, EPI_ISL_20182587–EPI_ISL_20182593, EPI_ISL_20182596, EPI_ISL_20182597, EPI_ISL_20195340, EPI_ISL_20195341. A(H3N2): EPI_ISL_20184088, EPI_ISL_20184090, EPI_ISL_20184238, EPI_ISL_20184245- EPI_ISL_20184247, EPI_ISL_20184250, and EPI_ISL_20184259-EPI_ISL_20184284. B/Victoria: EPI_ISL_20181505–EPI_ISL_20181528, EPI_ISL_20195342, EPI_ISL_20195343.

## Supplementary Materials

The following supporting information can be downloaded at: https://www.mdpi.com/article/10.3390/v18010013/s1, Table S1: Primers and probes used for cycling probe real-time PCR for B/Victoria; Table S2: Primers used for Sanger sequencing for B/Victoria; Figure S1: Clinical course of influenza B/Victoria infection with PA/I38T substitution in a family cluster. Timeline of illness and treatment among three siblings (ages 6, 10, and 13 years) in February 2024; Figure S2: Estimated prescription volume of anti-influenza drugs in Japan during the 2023–2024 season.
