# Supplementary material for: Influenza PA Substitutions and Genetic Diversity of A(H1N1)pdm09, A(H3N2), and B/Victoria Viruses in Japan During the 2023–2024 Season
Source: Viruses. 2025 Dec 21;18(1):13. doi: 10.3390/v18010013 (PMC12846502; doi:10.3390/v18010013)
Supplement: Supplementary file 1 [file viruses-18-00013-s001.zip › viruses-4040334-supplementary.pdf]

viruses

MDPI

Supplementary Material

Influenza PA Substitutions and Genetic Diversity of A(H1N1)pdm09, A(H3N2), and B/Victoria Viruses in Japan during the 2023–2024 Season

Table S1. Primers and probes used for cycling probe real-time PCR for B/Victoria.

Primers and probes	Sequence (5′→3′)	Position
B/Victoria I38T Forward primer	CAATGGCAGAATTTAGTGAAG	59-79
B/Victoria I38T Reverse primer	AGCCAGATACTTGGGAGTC	306-324
B/Victoria I38T FAM probe	TGGACACAG(A)TGTT	111-120
B/Victoria I38T ROX probe	TGGACACAG(G)TGT	111-119

Table S2. Primers used for Sanger sequencing for B/Victoria.

Primers and probes	Sequence (5′→3′)	Position
B/Victoria I38T 1st Forward primer	TCCAGACTACAATAATACAAAAGG	26-49
B/Victoria I38T 1st Reverse primer	GTAGCTCCTCATTCCTTCAAA	667-687
B/Victoria I38T 2nd Forward primer	ACAAAAGGCCAAAAACACAATG	42-63
B/Victoria I38T 2nd Reverse primer	GCACTCTCCCTTTCCCTTCC	492-511

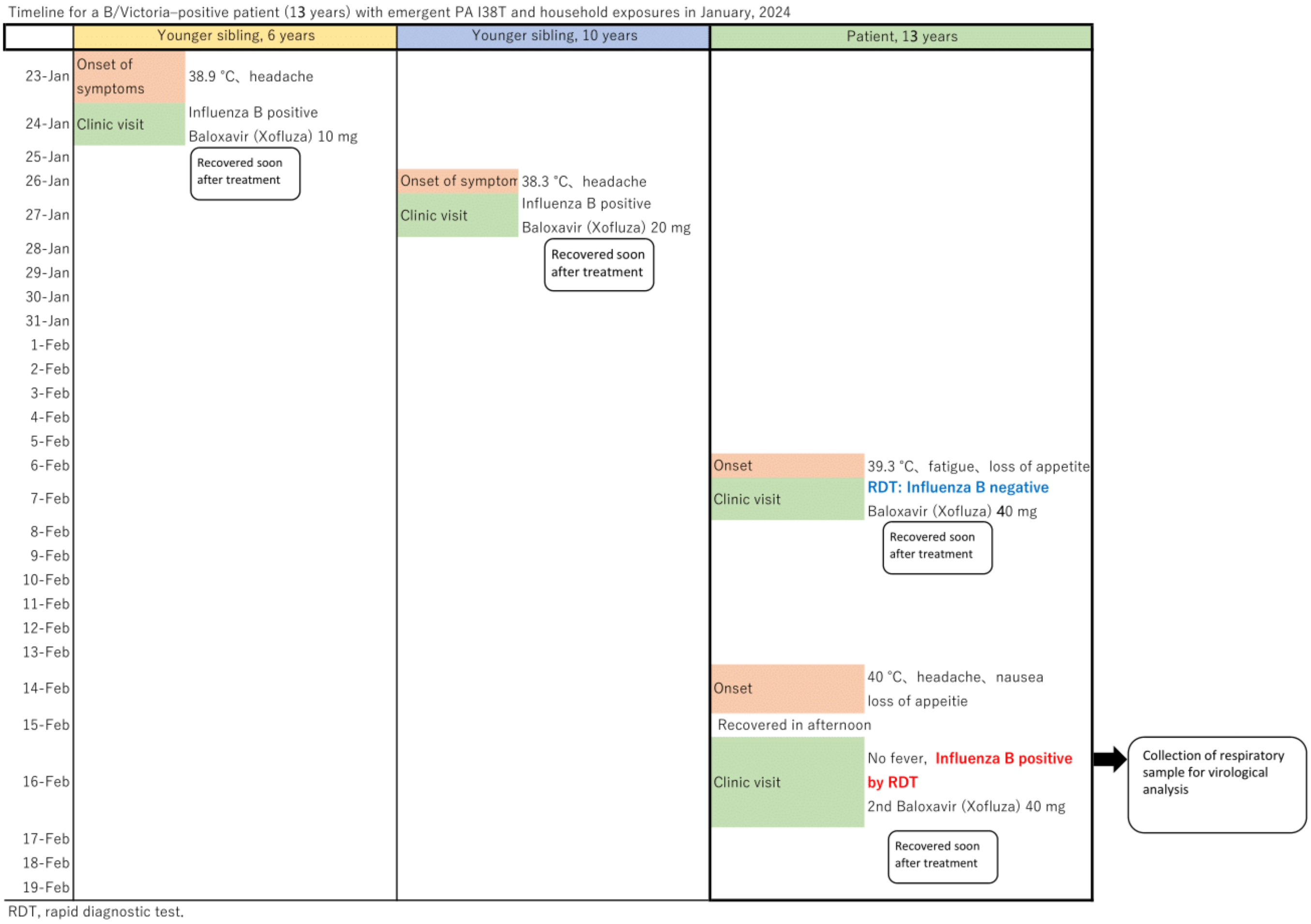

Figure S1. Clinical course of influenza B/Victoria infection with PA I38T substitution in a family cluster. Timeline of illness and treatment among three siblings (ages 6, 10, and 13 years) in February 2024. The 6- and 10-year-old developed influenza B infection and recovered rapidly following baloxavir treatment (first febrile episode). Two weeks later, the 13-year-old sibling experienced an initial febrile episode (6 February; peak 39.3 °C) treated empirically with baloxavir despite a negative RDT, followed by clinical improvement. A second, more severe febrile episode occurred on 15–16 February, confirmed by RDT, and a second dose of baloxavir was administered. A respiratory specimen collected at this second visit was negative by cycling-probe RT-PCR but yielded PA I38T substitution (61.1%) by NGS, despite wild-type sequence being called by Sanger sequencing.

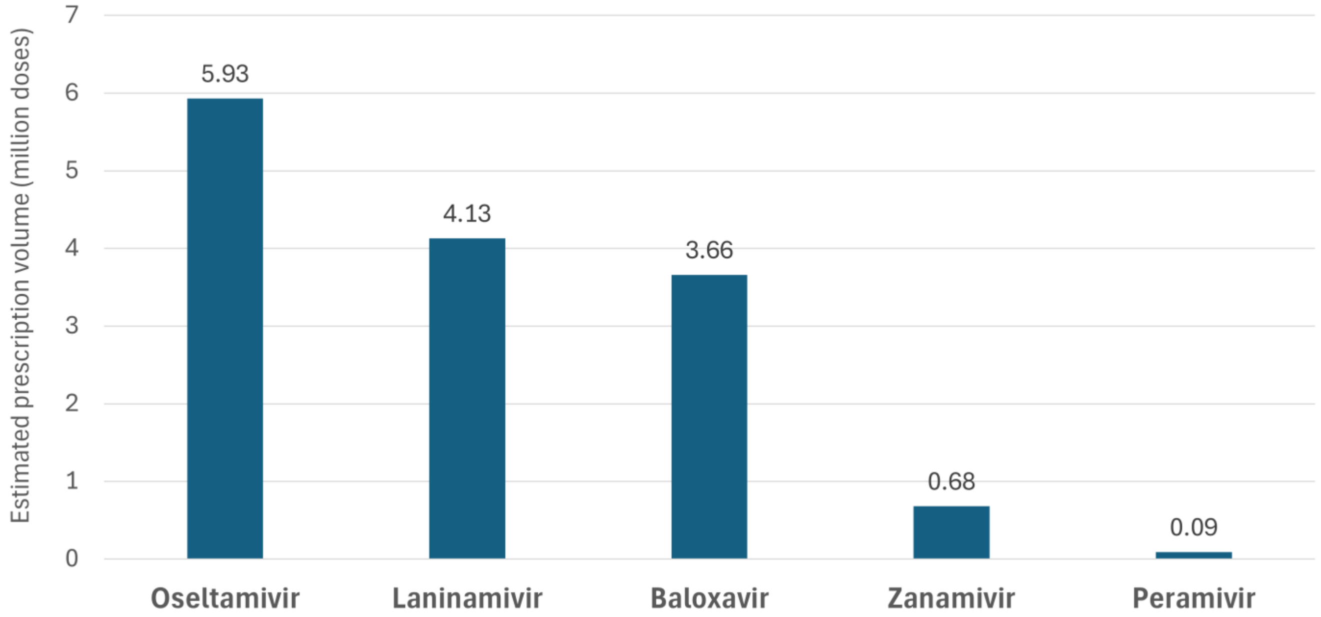

Figure S2. Estimated prescription volume and inventory of anti-influenza drugs in Japan during the 2023–2024 season. A) Estimated prescription volume (million doses) of anti-influenza drugs based on data from the Ministry of Health, Labour and Welfare, Japan. Oseltamivir was the most prescribed drug (5.93 million doses), followed by laninamivir (4.13 million), baloxavir (3.66 million), zanamivir (0.68 million), and peramivir (0.09 million).
